# Creutzfeldt–Jakob disease in a post-COVID-19 patient: did SARS-CoV-2 accelerate the neurodegeneration?

**DOI:** 10.1186/s41983-023-00666-y

**Published:** 2023-05-22

**Authors:** Taha K. Alloush, Adel T. Alloush, Yaser Abdelazeem, Hossam M. Shokri, Khaled O. Abdulghani, Ahmed Elzoghby

**Affiliations:** 1 Department of Neurology and Psychiatry, Faculty of Medicine, Ain Shams University, Cairo, Egypt; 2 Department of Geriatrics and Gerontology, Faculty of Medicine, Ain Shams University, Cairo, Egypt; 3 Departement of Diagnostic Radiology, Faculty of Medicine, Ain Shams University, Cairo, Egypt; 4 Department of Neurology and Psychiatry, Helwan University School of Medicine, Cairo, Egypt

**Keywords:** Sporadic Creutzfeldt–Jakob disease (sCJD), SARS-CoV-2, COVID-19, PET/MRI brain, Neurodegenerative disorder

## Abstract

**Background:**

Creutzfeldt–Jakob disease (CJD) is a rare, fatal neurodegenerative disorder, with few months as a usual duration from onset to death.

**Case presentation:**

In this case report, a patient of Sporadic CJD (sCJD) who presented one month after severe acute respiratory syndrome coronavirus-2 (SARS-CoV-2) infection. The diagnosis of this case was established after confirming findings from clinical, neurophysiology, radiological, and laboratory features of this disease.

**Conclusion:**

Putting in mind all the updated data on the pathogenesis of CJD and the immune responses to SARS-CoV-2, we can suggest that COVID-19 can lead to accelerated pathogenesis and exaggerated manifestations of this fatal neurodegenerative disease.

## Background

Creutzfeldt–Jakob disease (CJD) is a rare degenerative cause of rapidly progressive neurological dysfunction, affecting about one in every 1 million persons every year worldwide [1]. There are four described types of CJD; sporadic, familial, iatrogenic, and variant. Sporadic CJD (sCJD) is the most common form and accounting for 85% of CJD cases [2]. Early diagnosis, despite the heterogeneity of symptoms in early stages of the disease, is critical for the patient and their family members, to achieve optimal management and to dodge the chances of iatrogenic transmission [3, 4].

The pathophysiology behind CJD is the accumulation of abnormal PrP^Sc^ proteins in the central nervous system [5]. Usually, CJD appears in 55–75-year-old patients, with mortality of about 90% of cases over 1 year of the illness [6]. Myoclonus is the hallmark feature of CJD, yet it can present with behavioral changes, amnesia, and gait instability. In accordance with recent clinical diagnostic criteria for CJD, parietal, occipital, and temporal cortical regions are the most affected areas in MRI imaging. Hyperintensities in at least two of these areas are pathognomonic as well as sharp-wave complexes on the electroencephalography (EEG) or detection of 14-3-3 protein in the cerebrospinal fluid (CSF) [7].

On the other hand, the neurological effect of SARS-CoV-2 is not well-explained. Neurological manifestations of SARS-CoV-2 may include anosmia, headache, ischemic stroke, delirium, and encephalitis [8]. Besides the current evidence that SARS-CoV-2 can infect the nervous system directly, there are also suggested mechanisms for indirectly mediated inflammatory affection the nervous system [9, 10]. Moreover, the storming release of pro-inflammatory cytokines as IL-1, IL-6, IL12, and TNFα is suggested to cause neuroinflammation and can accelerate neurodegeneration process [11–16].

Here, the reported case demonstrates the clinical, radiological and laboratory features of sCJD in a post COVID-19 patient, with very rapid deterioration. This draws attention to some of the challenges faced during diagnosing patients with such rare neurodegenerative disease, and how these have been exaggerated by COVID-19.

## Case presentation

A 73-year-old previously healthy man was complaining of fever, cough and loss of smell on the 18th of March 2021. The lung CT scan, lymphopenia, and the positive result of SARS-CoV-2 PCR test confirmed the diagnosis of COVID-19. For which he received the recommended home therapeutic protocol with marked improvement.

**Fig. 1:**
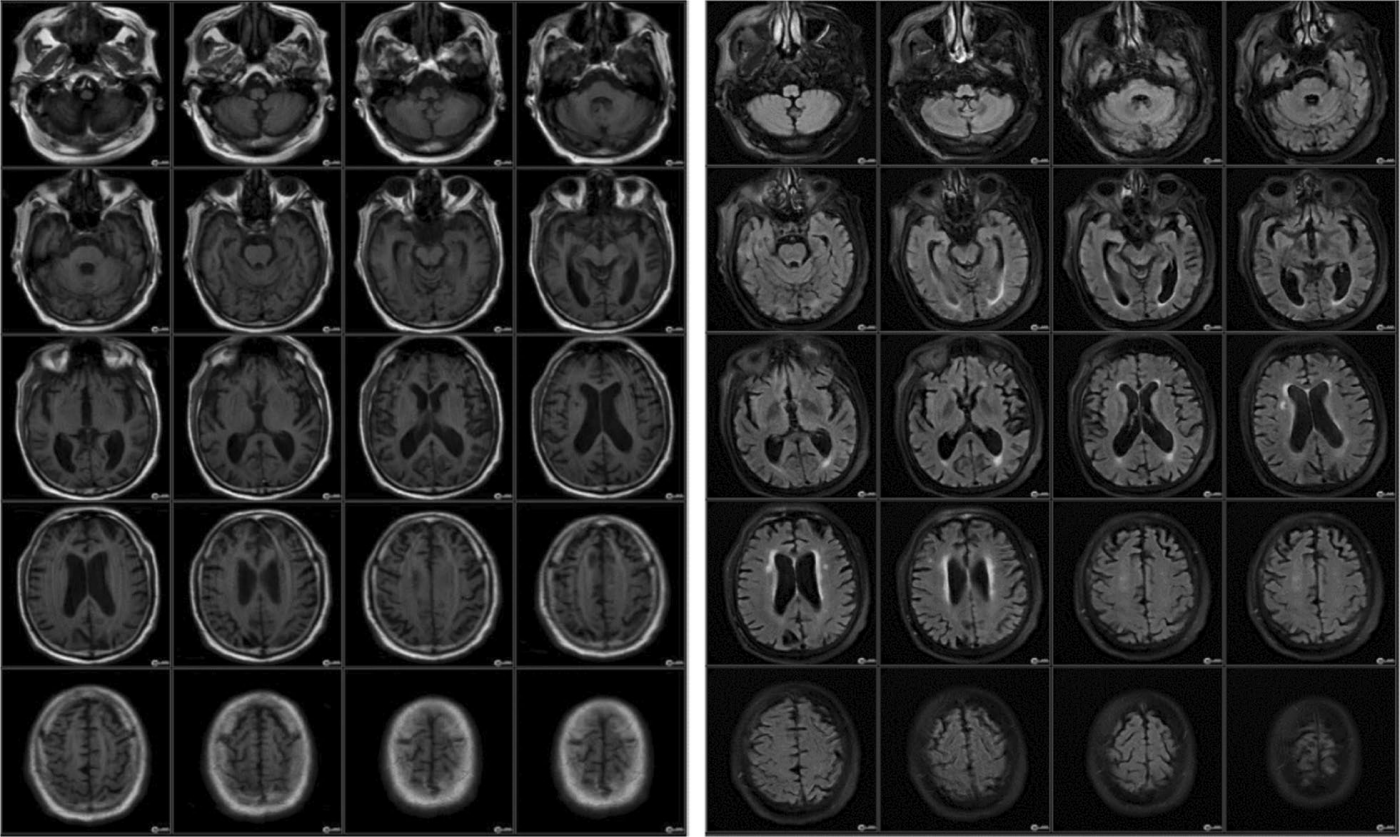
MRI brain axial T1 and FLAIR showing mild periventricular leukoencephalopathy, bilateral fronto-parietal small infarcts and central and to less extant cortical cerebral involutional changes

**Fig. 2:**
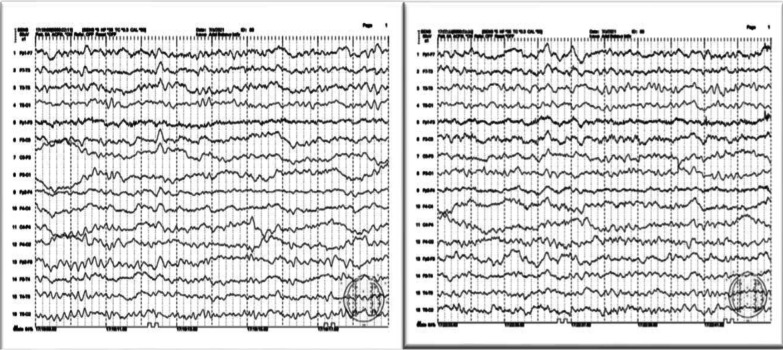
Abnormal EEG record showing moderate diffuse cerebral slowing

One month later, he started to complain of inattention, recent memory impairment, depressed mood, urinary incontinence and gait instability. Routine blood tests were normal, and a second SARS-CoV-2 PCR test was negative. Brain MRI showed mild periventricular leukoencephalopathy, bilateral fronto-parietal small infarcts at different stages of evolution, with central and, to a lesser extent, cortical cerebral involutional changes that raised the possible diagnosis of normal pressure communicating hydrocephalus (Fig. 1). However, CSF flowmetry study was done and showed no radiological evidence of hyperdynamic flow across the aqueduct. In addition, CSF analysis was acellular, with normal opening pressure, normal proteins and glucose levels with no bacterial growth in culture, negative HSV–PCR, tumor markers and serum autoimmune encephalopathy panels. EEG showed abnormal bilateral symmetrical and synchronous moderately diffuse cerebral slowing of 4–5 Hz; mixed delta–theta activity (Fig. 2).

**Fig. 3:**
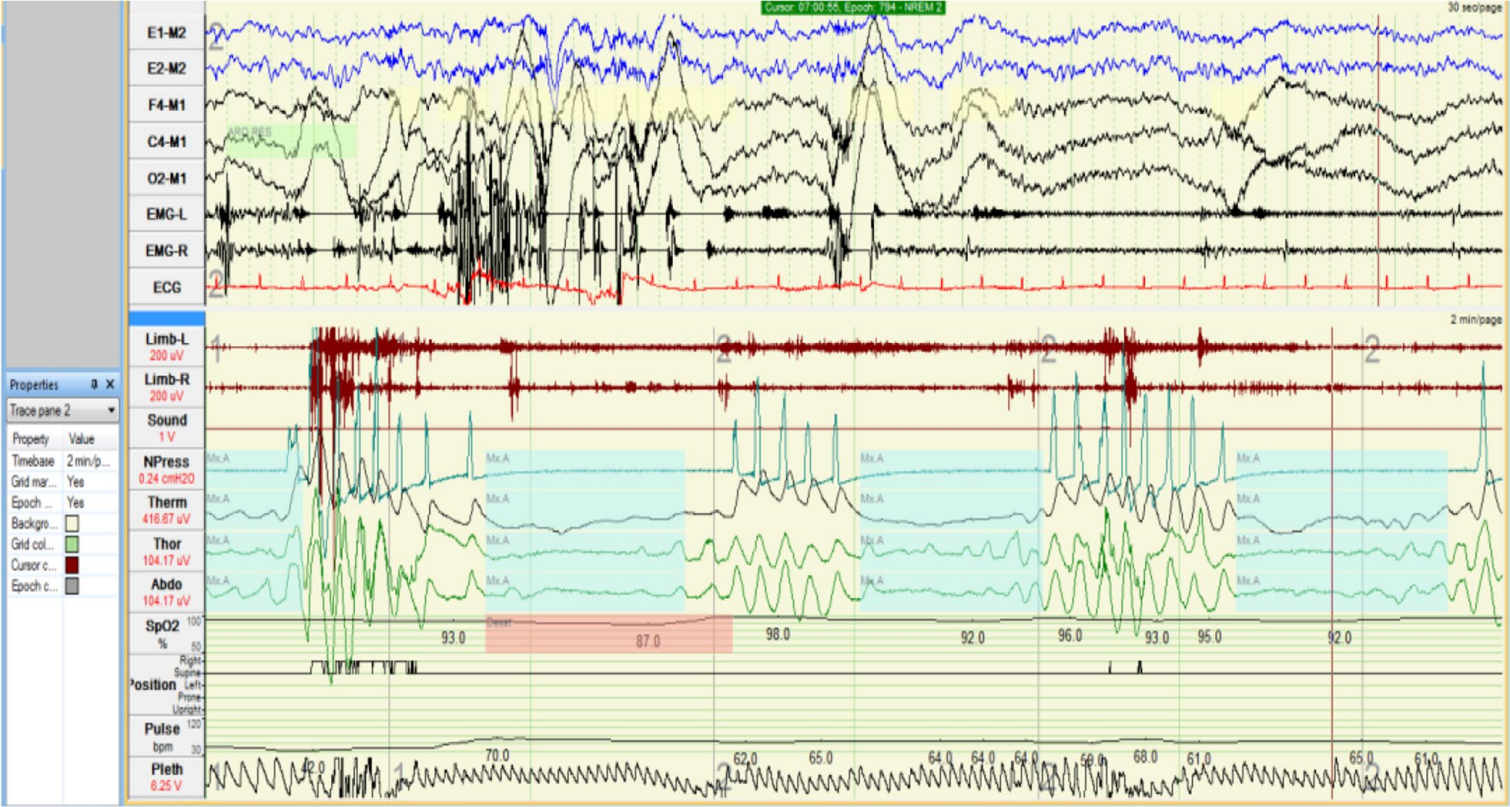
Polysomnography showing severe degree of obstructive sleep apnea

The patient’s condition deteriorated rapidly, as he became confused, disorientated to place with excessive daytime sleepiness, restless sleep, and loud snoring with periods of silence followed by gasps. Polysomnography was done and showed sleep efficiency of 74.5%, reduced REM and absent slow-wave sleep with apnea–hypopnea index of 36.3 events/hour, average awake SpO_2_ was 94% with lowest SpO_2_ 80% during periods of apnea during sleep. A diagnosis of severe obstructive sleep apnea was suggested that might be attributed to COVID-19 illness (Fig. 3), yet BiPaP ventilation trail showed no significant improvement. The patient’s condition deteriorated progressively, as he failed to recognize his family members, with anomia, poor comprehension, executive dysfunction, and recurrent attacks of intermittent diffuse myoclonic jerks.

**Fig. 4:**
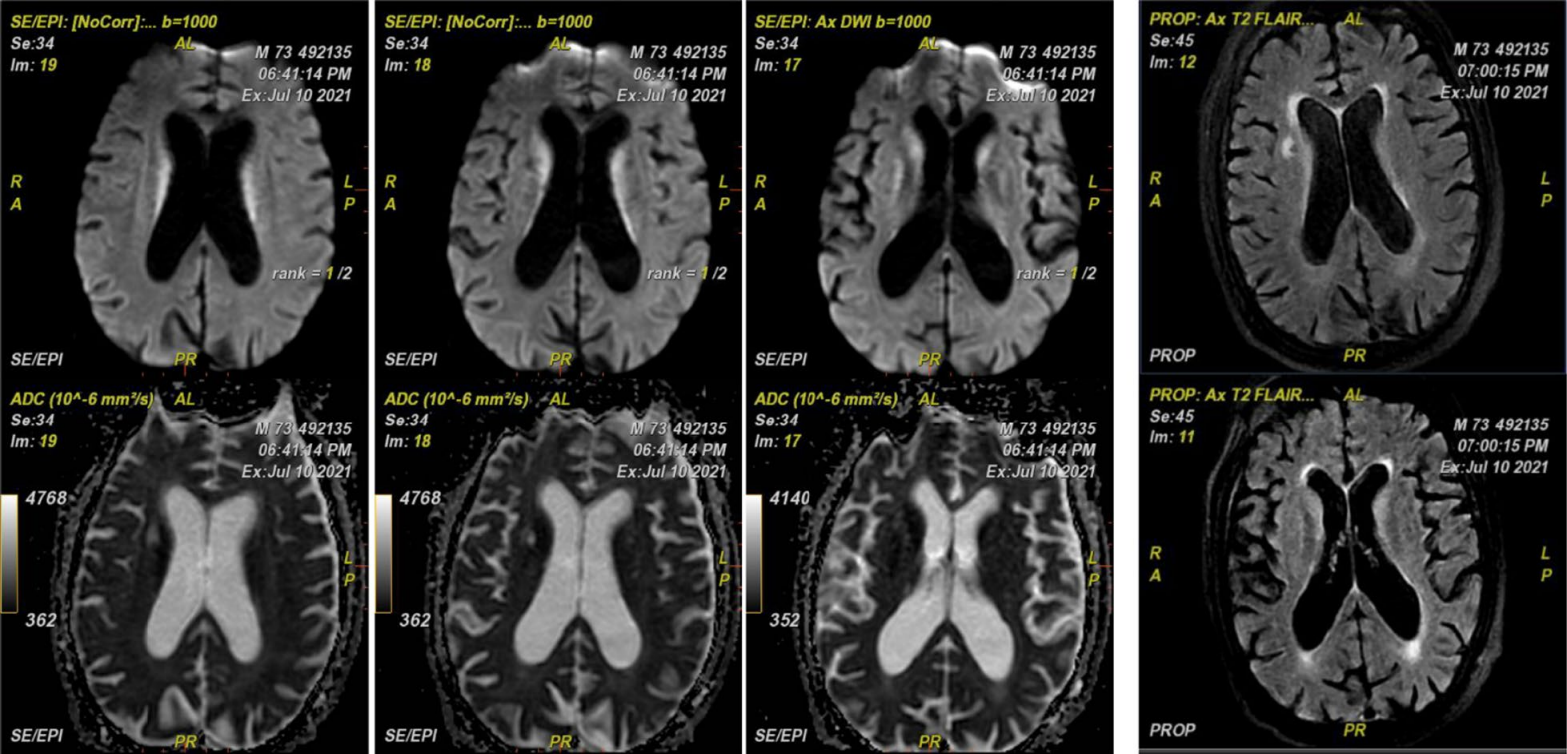
Conventional MRI data with diffusion images showing abnormal relative diffusion restriction with faint abnormal high signal on FLAIR, at the deep gray matter nuclei particularly caudate nuclei and anterior aspects of the putamen and thalamic nuclei

**Fig. 5:**
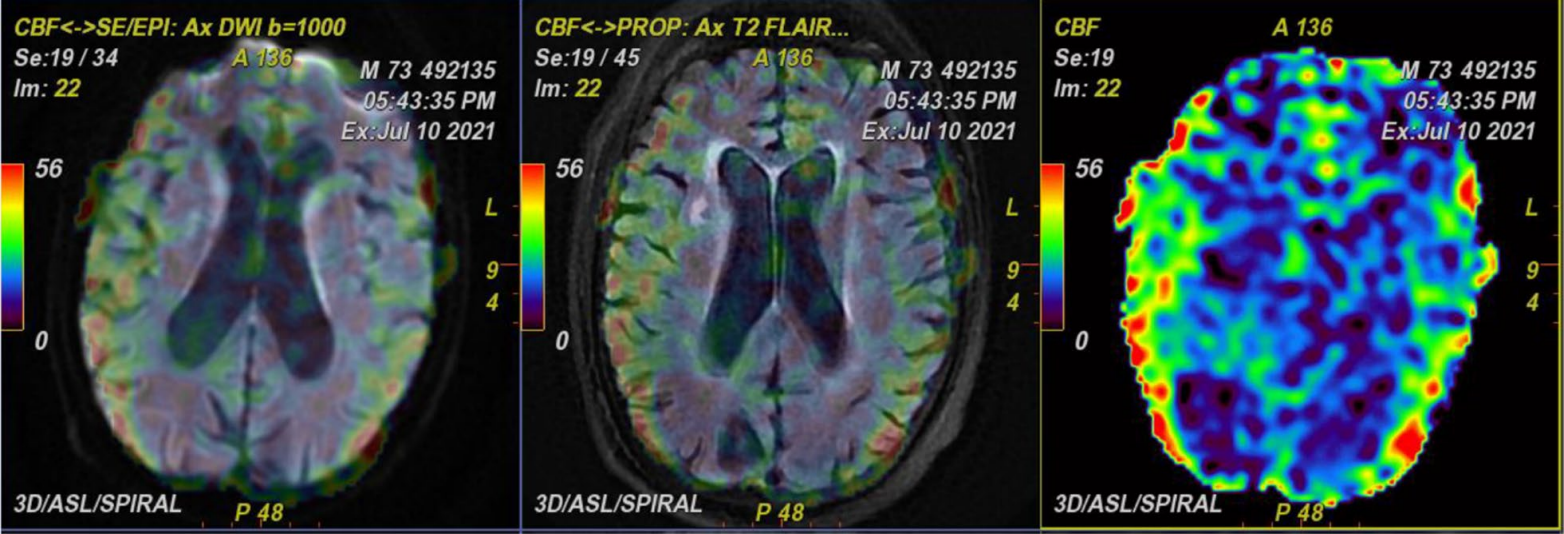
MR perfusion images showing mild hypoperfusion status of the deep cerebral white matter and deep gray matter bilaterally but with of normal perfusion of hippocampal formations and the precuneus regions

**Fig. 6:**
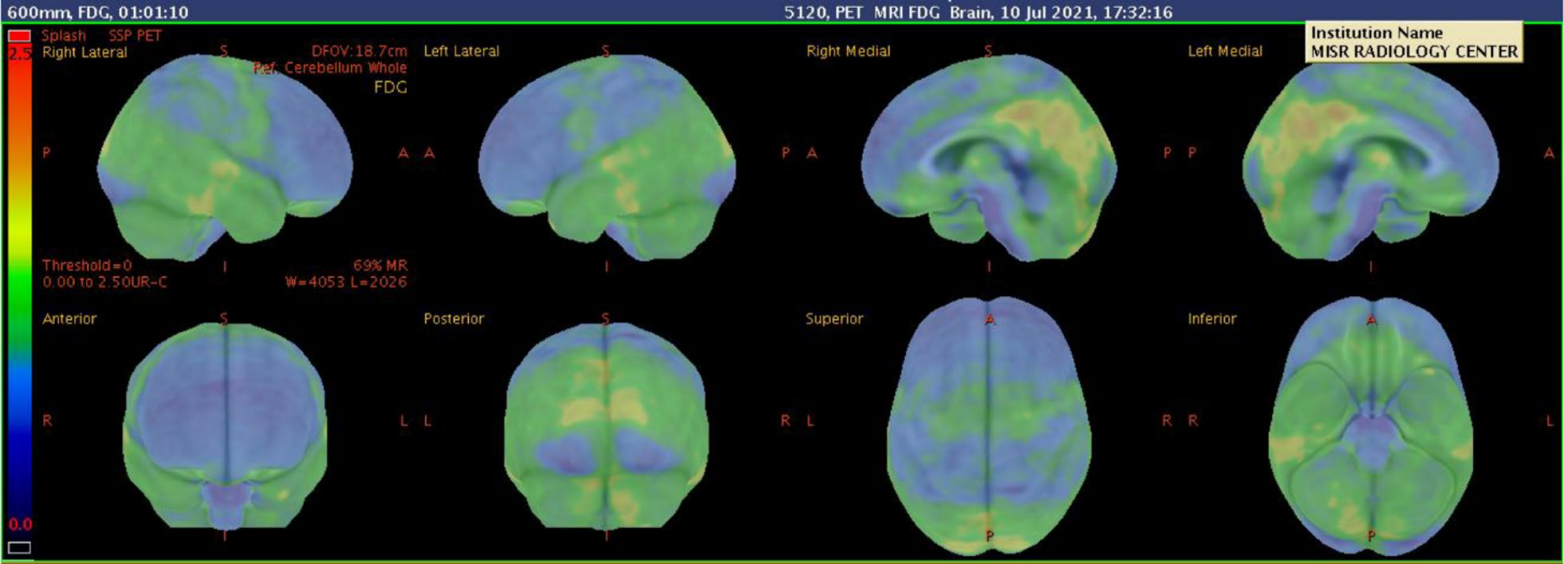
PET/MR data showing hypometabolic status seen regarding the cortical regions particularly at the right and left lateral prefrontal with sparing of the precuneus and posterior cingulum and occipital lobes and temporal lobes

**Fig. 7:**
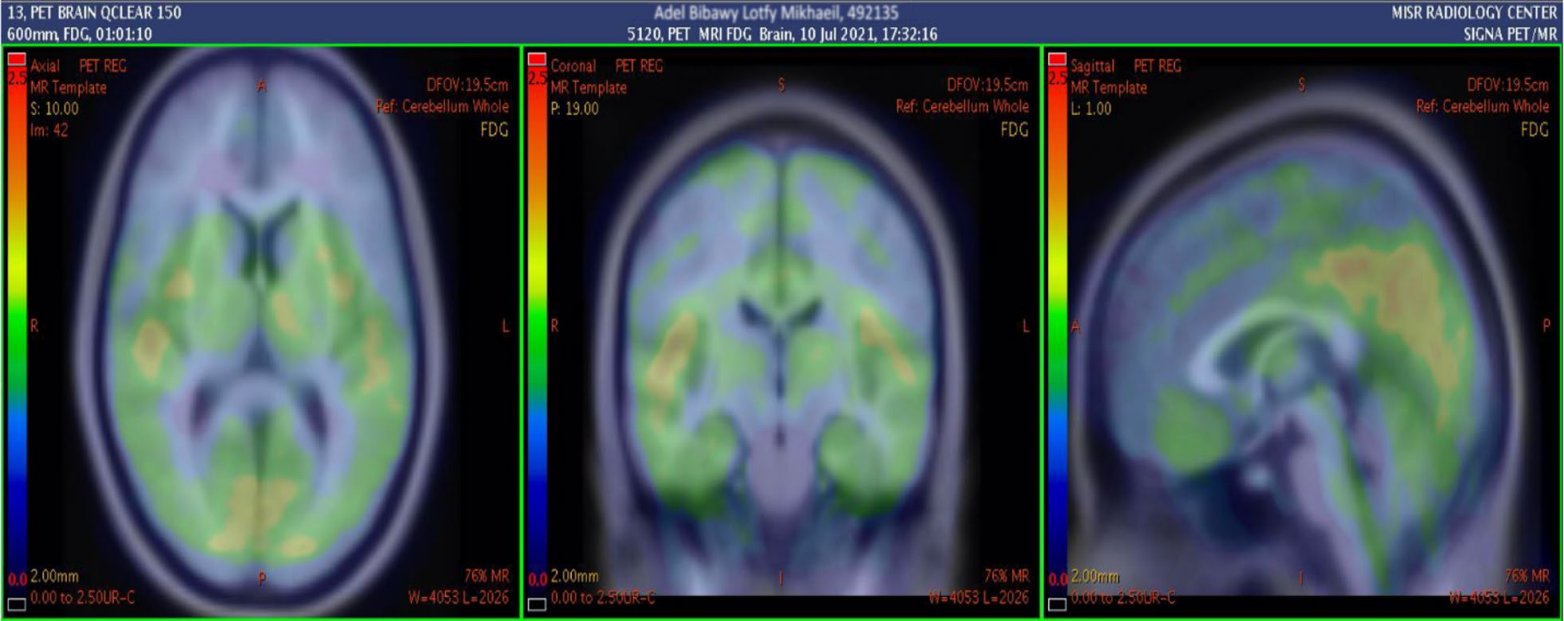
PET/MR data showing hypometabolic status of right and left medial prefrontal regions

Multi-parametric ^18^F-FDG hybrid PET/MRI brain scan revealed abnormal relative diffusion restriction at the deep gray matter nuclei, particularly the caudate nuclei and the anterior aspects of the putamen and thalamic nuclei (Fig. 4). These findings are mostly suggestive of CJD, particularly the sporadic form, and also ruled out COVID-19-related encephalopathy, as the later would have pronounced white matter affection deeply and to a lesser extent superficially. Moreover, MR perfusion data showed mild hypoperfusion status of the deep cerebral white matter and deep gray matter bilaterally. The hippocampal formations and the precuneus regions are of normal perfusion for this age, which can more or less rule out Alzheimer's disease diagnosis (Fig. 5). PET/MR data showed an evident hypometabolic status seen regarding the cortical regions particularly at the bilateral lateral prefrontal and to a lesser extent bilateral medial prefrontal regions. The precuneus, posterior cingulum, occipital lobes and temporal lobes were spared from such hypometabolism ruling out other causes of dementia like Lewy body dementia (Figs. 6, 7).

CSF 14-3-3 protein assay was ordered revealing a positive result. During hospitalization, the patient was treated with high doses of corticosteroids before the diagnosis of sCJD was suggested, but there was no significant improvement. Unfortunately, his neurologic status progressed to mutism, vegetative state, and coma. Sadly, he died three months after the symptoms’ onset.

Since the development of COVID-19 pandemic, there have been many reports of neurological insults directly related to the SARS-CoV-2 infections. While cerebrovascular strokes seem to be the most frequent neurological sequalae, encephalopathies, encephalitis and neuropsychiatric disorders have been also reported [8, 17]. COVID-19 was our first suggested diagnosis for our patient; however, this suggestion was excluded following the negative result of his SARS-CoV-2 PCR test, supported with the absence of specific brain MRI findings observed in patients with COVID-19 as diffuse leukoencephalopathy, restricted diffusion and juxtacortical and callosal microhemorrhages [18].

The sCJD is usually fatal within months, yet about 15% of sCJD patients survive for more than two years [6]. In our case presented here, the rapid deterioration, as the patient died just four months after the onset of the disease, raises the suspicion of the presence of additional precipitating factor. Generally in prion disease, reactive A1 astrocytes are believed to have a neurotoxic effect and act as foci for PrP^Sc^ propagation [19]. The concurrence of CJD in this post-COVID-19 patient has directed us to a hypothesis that the systemic inflammatory mediators storm occurring with COVID-19 may have accelerated the CJD pathogenesis and hence the rapid progression of neurodegeneration. There is an observational support for this hypothesis, as the inflammatory storm occurring in COVID-19 releases massive amounts of inflammatory mediators that are enough for activation A1 astrocytes which is directly related to PrP^Sc^ propagation as stated earlier [20–22].

Two case reports recently concluded that there is a sudden surge in the systemic inflammatory response due to COVID-19 and concluded the same hypothesis of accelerated progression of sCJD cases [23–25].

## Conclusions

When a patient of sCJD gets infected with SARS-CoV-2, the neurological deterioration gets accelerated, exacerbated, and consequently shortening the overall survival period. Therefore, extra-vigilance and intensive monitoring for such patients is highly recommended.

## Data Availability

The data sets generated and analyzed during the current study are not publicly available due to institutional limitations, yet they are available from the corresponding author on reasonable request.

## Abbreviations

BiPaP: Biphasic positive airway pressure
CJD: Creutzfeldt–Jakob disease
COVID-19: Coronavirus disease 2019
CSF: Cerebrospinal fluid
EEG: Electroencephalography
HSV: Herpes simplex virus
IL: Interleukins
PCR: Polymerase chain reaction
PrP^Sc^: Scrapie isoform of the prion protein
SARS-Cov-2: Severe acute respiratory syndrome coronavirus-2
sCJD: Sporadic Creutzfeldt–Jakob disease
TNF: Tumor necrosis factor

## References

[1] Uttley L, Carroll C, Wong R, Hilton DA, Stevenson M (2020). Creutzfeldt-Jakob disease: a systematic review of global incidence, prevalence, infectivity, and incubation. Lancet Infect Dis.

[2] Manix M, Kalakoti P, Henry M, Thakur J, Menger R, Guthikonda B (2015). Creutzfeldt-Jakob disease: updated diagnostic criteria, treatment algorithm, and the utility of brain biopsy. Neurosurg Focus.

[3] Mead S, Rudge P (2017). CJD mimics and chameleons. Pract Neurol.

[4] Carswell C, Thompson A, Lukic A, Stevens J, Rudge P, Mead S (2012). MRI findings are often missed in the diagnosis of Creutzfeldt-Jakob disease. BMC Neurol.

[5] Sikorska B, Knight R, Ironside JW, Liberski PP (2012). Creutzfeldt Jakob disease. Adv Exp Med Biol..

[6] Vacca VM (2016). CJD: understanding Creutzfeldt-Jakob disease. Nursing.

[7] Zerr I, Kallenberg K, Summers DM, Romero C, Taratuto A, Heinemann U (2009). Updated clinical diagnostic criteria for sporadic Creutzfeldt–Jakob disease. Brain.

[8] Wu Y, Xu X, Chen Z, Duan J, Hashimoto K, Yang L (2020). Nervous system involvement after infection with COVID-19 and other coronaviruses. Brain Behav Immun.

[9] Mesci P, Macia A, Saleh A, Martin-Sancho L, Yin X, Snethlage C (2020). Sofosbuvir protects human brain organoids against SARS-CoV-2. bioRxiv.

[10] Yachou Y, El Idrissi A, Belapasov V, Ait BS (2020). Neuroinvasion, neurotropic, and neuroinflammatory events of SARS-CoV-2: understanding the neurological manifestations in COVID-19 patients. Neurol Sci.

[11] Bright F, Werry EL, Dobson-Stone C, Piguet O, Ittner LM, Halliday GM (2019). Neuroinflammation in frontotemporal dementia. Nat Rev Neurosci.

[12] Holmes C, Cunningham C, Zotova E, Woolford J, Dean C, Kerr S (2009). Systemic inflammation and disease progression in Alzheimer disease. Neurology.

[13] Tan EK, Chao YX, West A, Chan LL, Poewe W, Jankovic J (2020). Parkinson disease and the immune system—associations, mechanisms and therapeutics. Nat Rev Neurol.

[14] Hoffmann A, Ettle B, Battis K, Reiprich S, Schlachetzki JCM, Masliah E (2019). Oligodendroglial α-synucleinopathy-driven neuroinflammation in multiple system atrophy. Brain Pathol.

[15] Khandelwal PJ, Herman AM, Moussa CE-H (2011). Inflammation in the early stages of neurodegenerative pathology. J. Neuroimmunol..

[16] Stoeck K, Schmitz M, Ebert E, Schmidt C, Zerr I (2014). Immune responses in rapidly progressive dementia: a comparative study of neuroinflammatory markers in Creutzfeldt-Jakob disease, Alzheimer's disease and multiple sclerosis. J Neuroinflam.

[17] Varatharaj A, Thomas N, Ellul MA, Davies NWS, Pollak TA, Tenorio EL (2020). Neurological and neuropsychiatric complications of COVID-19 in 153 patients: a UK-wide surveillance study. Lancet Psychiatry.

[18] Radmanesh A, Derman A, Lui YW, Raz E, Loh JP, Hagiwara M (2020). COVID-19-associated diffuse leukoencephalopathy and microhemorrhages. Radiology.

[19] Makarava N, Chang J-C-Y, Molesworth K, Baskakov IV (2020). Region-specific glial homeostatic signature in prion diseases is replaced by a uniform neuroinflammation signature, common for brain regions and prion strains with different cell tropism. Neurobiol Dis..

[20] Liddelow SA, Guttenplan KA, Clarke LE, Bennett FC, Bohlen CJ, Schirmer L (2017). Neurotoxic reactive astrocytes are induced by activated microglia. Nature.

[21] Cavalli G, De Luca G, Campochiaro C, Della-Torre E, Ripa M, Canetti D (2020). Interleukin-1 blockade with high-dose anakinra in patients with COVID-19, acute respiratory distress syndrome, and hyperinflammation: a retrospective cohort study. Lancet Rheumatol.

[22] Merad M, Martin JC (2020). Pathological inflammation in patients with COVID-19: a key role for monocytes and macrophages. Nat Rev Immunol.

[23] Young MJ, O’Hare M, Matiello M, Schmahmann JD (2020). Creutzfeldt-Jakob disease in a man with COVID-19: SARS-CoV-2-accelerated neurodegeneration?. Brain Behav Immun.

[24] McMurran CE, Chaggar GH (2020). Ugoya SO (2020) A patient with sporadic Creutzfeldt Jakob disease: challenges of rare diseases in the COVID-19 era. Oxf Med Case Rep.

[25] Choudhary S, Sonkar M, Saxena AK (2021). A case report of sporadic Creutzfeldt-Jakob disease in an Asian origin coronavirus disease-19 patient: an enigma. Indian J Case Rep.

